# Reproductive Performance, Inbreeding, and Genetic Diversity in Montbeliarde Dairy Cattle Obtained by Absorption Crossing

**DOI:** 10.3390/ani15030322

**Published:** 2025-01-23

**Authors:** Luis F. Cartuche-Macas, Oscar J. Guaman Ilvay, Edilberto Chacón, Miguel A. Gutierrez-Reinoso, Manuel Garcia-Herreros

**Affiliations:** 1 Asociación Holstein Friesian del Ecuador (AHFE), Quito 170802, Ecuador; 2 Facultad de Ciencias Agropecuarias y Recursos Naturales, Carrera de Medicina Veterinaria, Universidad Técnica de Cotopaxi, Latacunga 050101, Ecuador; 3 Laboratorio de Biotecnología Animal, Departamento de Ciencia Animal, Facultad de Ciencias Veterinarias, Universidad de Concepción (UdeC), Chillán 3780000, Chile; 4 Instituto Nacional de Investigação Agrária e Veterinária (INIAV), 2005-424 Santarém, Portugal; 5 CIISA-AL4AnimalS, Faculty of Veterinary Medicine, University of Lisbon, 1300-477 Lisbon, Portugal

**Keywords:** endogamy, population dynamics, reproductive parameters, diversity loss, Montbeliarde, dairy cattle

## Abstract

Montbeliarde dairy cattle were introduced in Ecuador with the aim of ameliorating the production performance of other cattle breeds. The aim was to analyze the reproductive performance, demographic structure, inbreeding, and genetic diversity of Montbeliarde cattle obtained by absorption crossing. Official pedigree records from the Ecuadorian Montbeliarde Association were used. The total population was divided into seven populations: (i) historical (all individuals), (ii) 1999–2003, (iii) 2004–2008, (iv) 2009–2013, (v) 2014–2018, (vi) 2019–2023 (current), and (vii) reference (individuals with known parents). Demographic structure variables analyzed: pedigree completeness index (PCI), number of equivalents (GEqu), complete (GCom) and maximum (GMax) generations, and generation interval (GI). Inbreeding-derived variables analyzed: inbreeding (F), inbreeding increment (ΔF), average relatedness (AR), co-ancestry (C), non-random mating (α), effective population size (Ne), and genetic conservation index (GCI). Gene origin probability/genetic diversity variables analyzed: number of founders (f), effective number of founders (fe) and ancestors (fa), number of equivalent genomes (fg), fe/fa and fg/fa ratio, and DG losses. The databases were analyzed by ENDOG, POPREP, and CPC software. PCI in the historical population compared to the current population decreased from 72.95 to 65.87% (sire/dam pathway), while the GI decreased from 7.17 to 3.08 years when the historical and current populations were compared. Dam reproductive efficiency increased over time. Moreover, F = ~1%, AR = 2.06%, ΔF = 0.22%, C = 1.03%, α = −0.0077, GCI = 3.12, and Ne = 58 values were obtained. Gene origin probability in the reference population was f = 439, fa = 37, fe = 71.64, fg = 23.43, and fg/fe = 0.33, showing a GD loss due to unequal contribution of founders (0.55%) and bottleneck and genetic drift (4.25%). In conclusion, the Ecuadorian Montbeliarde cattle population displayed a relatively low diversity and high genetic relationship. Inbreeding levels increased while Ne values decreased over time. The introduction of new purebred bloodlines could be important to minimize the inbreeding levels ensuring the long-term conservation of this breed and minimizing GD loss.

## Simple Summary

Although breeding and selection schemes improve genetic gains, it appears to also result in higher levels of inbreeding, which may adversely impact the genetic diversity of dairy cattle populations. The present study was designed to analyze the reproductive performance, population structure, inbreeding, and genetic diversity of Ecuadorian Montbeliarde cattle obtained by absorption crossing. It was concluded that inbreeding rates and genetic diversity losses increased linearly over time, being very close to critical levels, which could result in negative effects on the conservation purposes of Montbeliarde dairy cattle.

## 1. Introduction

The introduction of dairy cattle breeds as a genetic improvement strategy has been a constant in Ecuador. Currently, there have been at least 17 dairy cattle breeds introduced in the country [1,2], of which only the Holstein Friesian, Jersey, Brown Swiss, Simmental, Normande, Gyr-Girolando, and Montbeliarde breeds have a breeders’ association that keeps the genealogical records and, in some cases, the dairy monitorization [3,4,5,6]. The strategies for introducing the different dairy cattle breeds have been mainly to take advantage of hybrid vigor (crosses with Holstein Friesian), adaptability to the altitude of the Ecuadorian highlands, and to improve the quality of milk- and dairy-derived products for human consumption [7]. In the 1990s, the Montbeliarde breed was introduced from France through the importation of artificial insemination (AI) doses [8]. One of the main reasons was related to the fact that the milk produced by the Montbeliarde breed presents high quality in cheese dry matter (higher fat:protein ratio). Other reasons include adaptation to dry forage, the muscular quality of individuals, more profitable offspring, resistance to large thermal amplitudes, good fertility, and extensive longevity, in addition to the fact that crossbreeding with other dairy breeds can improve their resistance and fertility [9]. Over the years, some cattle producers became interested in the Montbeliarde breed and began their selection and breeding scheme through the absorption crossing method due to the existing difficulty in importing embryos from Europe and the high costs of the embryo transfer technique, among other reasons [10]. Currently, this breed is being bred and selected for various production systems, both for crossbreeding and purebred schemes [11]. Lately, the Montbeliarde breed is even being used in sustainable production systems, considering the animal-plant-soil-water balance [12].

Nowadays, the regulation of the Ecuadorian Ministry of Agriculture, Livestock, Aquaculture, and Fisheries permits the importation of live animals, semen, and frozen embryos for genetic improvement of this breed [13]. Imported genetic material must come from individuals belonging to countries with solid genetic improvement programs, meaning that they possess genetic values with a high degree of reliability [14,15]. In addition, during the last decade (since the 2010s), the importation of live animals analyzed by genomic evaluations has been initiated in order to improve the genetic progress of this breed in Ecuador [16]. Similarly, the Ecuadorian Montbeliarde Association has managed the studbook since its formation (90s) in order to improve this breed [17]. In other dairy breeds, the genealogical data have been used for the evaluation of genetic diversity, population structure, and gene flow, but in the case of the Ecuadorian Montbeliarde, no studies have been carried out to date, with the particularity that this breed has been formed from a selection and breeding scheme through the method of absorption crossing [18]. In addition, at present, this breed has a genetic improvement plan in which the kinship of the individuals in the crossing and selection schemes is being evaluated. However, due to the origin of selection and breeding of this breed in Ecuador, there could be a genetic diversity loss over time, as well as an increase in inbreeding rates at the individual and population level, which could negatively influence the future of this breed [19,20,21].

Thus, the objective of the present study was to analyze the genetic diversity and population structure of the Montbeliarde breed whose origin in Ecuador was created from a selection and breeding scheme using the absorption crossing method. Parameters such as inbreeding, reproductive performance, generation interval, coancestry, average kinship, probability of gene origin, and the different Wright-derived statistics will be analyzed in order to design a genetic plan to improve the management of this breed and avoid possible genetic diversity loss.

## 2. Materials and Methods

### 2.1. Ethical Statement

The present research did not require any animal handling, since the study was directly carried out using the records and databases provided by the Ecuadorian Montbeliarde Association (ACM, Ecuador), the Institut de l’élevage (IDELE, France), and the Irish Cattle Breeding Federation (ICBF, Ireland).

### 2.2. Genealogical Database

The genealogical database was provided by the Ecuadorian Montbeliarde Association. A total of 2399 registered animals were used, including 366 bulls and 2033 cows, born until December 2023 (including genetic material from sires imported via AI doses). For the analysis, seven populations were considered: historical population (individuals born between 1969 and 2023) and five populations born from 1999 to 2023 taken at 5-year intervals (1999–2003; 2004–2008; 2009–2013; 2014–2018; 2019–2023), which included 211, 332, 747, 434, and 167 individuals, respectively. The seventh was the reference population, which was considered to be the one encompassing individuals with known sire and dam from the populations described above. Populations were defined because calculations related to genetic diversity, gene origin probabilities, and founder analyses can only be performed by considering only individuals with both parents known or by comparing them with historical and current data sets, as suggested by Casanovas Arias et al. [22] and Navas et al. [23]. ENDOG (v 4.8) software [24,25] was used to perform demographic and genetic analyses to quantify and trace genetic diversity back to ancestors.

### 2.3. Absorption Breeding Scheme

Within the process of the Montbeliarde breed establishment and genetic management, the “Pure Breed” (PB) genealogical herd book was established, including only individuals with DNA parentage proofs guaranteeing their genetic purity. Absorption crossing was selected as the best option to improve the purity of individuals since the importation of live animals was prohibited during the 60s. The absorption crossing consists of the achievement of a mating, at each generation, between the crossed females (at the first stage, pure Holstein Friesian females x pure Montbeliarde males) and purebred Montbeliarde males. The absorption crossing process improved the breed purity in favor of the Montbeliarde across generations, which resulted in a progressive increase in the percentage of purity genes of the Montbeliarde breed from one generation to another. The use of the crossed Holstein Friesian females (initial population) for the creation of the highest purity genotype increased the capacity of adaptation of the Montbeliarde breed to the new environmental conditions. The individuals of high genetic purity were obtained from crosses using the initial population. Thus, the first generation was generated (known parents; purity: 50%), then the second (known parents and grandparents; purity: 75%), third (known parents, grandparents, great grandparents; purity: 87.5%), and fourth (known parents, grandparents, great grandparents, and great-great grandparents; purity: >93%). Finally, only the fifth generation of females (purity: 31/32; >96%) were registered in the PB herd book. In all cases (across generations), the parental genetic material was obtained from artificial insemination doses from purebred (purity: 100%) males of French origin. Finally, after the absorption crossing scheme, the individuals were registered in the herd book, not only for having the required purity, but also for having the required phenotypic characteristics such as hair color and other zoomorphometric characteristics (linear evaluation >86). All linear classifications were carried out by an independent external committee.

### 2.4. Demographic Structure

First, the number of births was calculated in order to determine the maximum and average number of calves per sire/dam. The total and mated dam/sire ratio was also calculated by dividing the total number of dams by the total number of sires, as well as the number of breeding dams divided by breeding sires, respectively. In addition, the pedigree integrity index (PCI) was calculated following the assumptions of Navas et al. (2017) [23] from the first to the fifth generation, and also the number of maximum generations (GMax), number of complete generations (GCom), and number of equivalent generations (GEqu) in the five defined populations. Moreover, the generation interval (GI) was calculated for the 4 gametic pathways from sire and dam to son and daughter, respectively, according to James [26]. For this purpose, the record of the birth date of each individual together with that of its parents was used. In parallel, gene flow between herds was evaluated according to the contribution of sires to the population [27].

### 2.5. Reproductive Performance-Derived Traits

The evolution of the reproductive performance in the Montbeliarde breed population obtained by absorption crossbreeding was studied by dividing the whole period (1999–2023) into five periods, as previously stated. Based on the historical official records obtained from the Ecuadorian Montbeliarde Association, several parameters related to the reproductive performance were analyzed during the different chronological periods. The number of births obtained from the official records was analyzed to establish both the number of offspring per sire and dam as follows: (i) the average number of calves per sire; (ii) the maximum number of calves per sire; (iii) the average number of calves per dam; and (iv) the maximum number of calves per dam. Moreover, the average age of parents at the birth of offspring and generation intervals were recorded and analyzed using birth date records for each individual and their respective parents (sire and dam). In addition, the distribution of cows by calving number (calves/cow/year) was obtained. Finally, the influence of the number of services per calving based on the different chronological periods studied was analyzed.

### 2.6. Inbreeding and Coancestry

Inbreeding coefficient (F): The F was estimated using the algorithm proposed by Meuwissen and Luo [28], and the inbreeding increment (ΔF) per generation was calculated using the equation proposed by Gutiérrez et al. [25]. The F has been defined as the probability that two alleles taken at random are identical per offspring:

∆F=Ft−Ft−11−Ft−1

where Ft and Ft−1 are the average inbreeding of the tth generation (i = 1, …, t).

Average relatedness (AR): Average relatedness was defined as the probability that a randomly selected allele from a population belongs to a specific individual, which was calculated using the vector c, where each element corresponds to the respective AR of an individual, defined by Gutiérrez and Goyache [25]. Each individual’s average relatedness coefficient (AR) was defined as the probability that two related individuals have inherited a particular allele of a single locus/gene from their common ancestor (this allele is known as IBD: identical by descent):

c’ = (1⁄n)1’A

where “A” is the n × n parentage matrix.

Coancestry (C): The C between two individuals is the probability that the genes, taken at random from each of the individuals, are identical by descent [29]. As a result, the C between two individuals is the F of their potential offspring. The C between two individuals is equal to the inbreeding coefficient of their offspring if the individuals are related [30]. It was also used to analyze the degree of relatedness and nonrandom mating, α, within breeds. Coancestry was estimated according to a specific algorithm [31]. 

Individual coancestry rate (∆C): The ∆C for each generation was calculated following the methods described by Cervantes et al. [20]:

$$C_{ba}=1-\sqrt[{\frac{tb+ta}{2}}]{1-C_{ba}}$$

where tb and ta are the number of equivalent complete generations and Cba is the C for individuals b and a.

Non-random mating (α): Non-random mating indicates the degree of deviation from Hardy-Weinberg proportions and is related to the inbreeding coefficients according to Sheppard and Wright [32]. The α was estimated as the correlation of genes between two individuals in relation to the correlation of genes taken at random from the population (α) according to Caballero and Toro [33]:

$$\left(1-F\right)=\left(1-C\right)\left(1-\alpha \right)$$

Effective size (Ne): The effective size was calculated as proposed by Hill [34,35]. The Ne was defined as the number of males and females contributing to genetic variability in a population [32,36]:

Ne=12∆F

Three additional Ne values were also estimated using the regression coefficient (b) of the individual inbreeding coefficients on: (i) the full number of generations, (ii) the maximum number of generations, and (iii) the equivalent number of full generations, with the regression coefficient corresponding to the increment between the two inbreeding generations (Fn − Fn−1 = b) [24,34,35]:

Ne=12b

Genetic Conservation Index (GCI): The GCI was estimated from the genetic contribution of all founders, considering the proportion of genes from a founder animal in the pedigree under analysis according to Alderson [37]. The following equation was used:

GCI=1∑pi2

where “pi” is the proportion of genes of founder “i” in the individual’s pedigree.

### 2.7. Gene Origin Probabilities and Ancestral Contributions

Number of founders (f): The f was defined as those individuals with unknown parents, assumed to be unrelated and having an inbreeding coefficient of 0.

Effective number of founders (fe): The effective number of founders is estimated from the following equation [38]. The fe was defined as the number of founders that contribute equally and are expected to produce the same genetic diversity as the study population:

$$f_{e}=\frac{1}{\sum_{k=1}^{f}q_{k}^{2}}$$

where “qk” is the gene origin probability from ancestor “k and “f” is the real number of founders.

Effective number of ancestors (fa): The fa was defined as the minimum number of ancestors that are not necessarily founders and that account for the full genetic diversity of a population [39]:

$$f_{a}=\frac{1}{\sum_{k=1}^{f}p_{k}^{2}}$$

where “pk” is the marginal contribution of an ancestor “k”, which is not explained by other chosen ancestors, and “f” is the real number of founders.

Number of founder genome equivalents (fg): This parameter was estimated from twice the inverse of the average C according to Caballero and Toro [33]. The fg was defined as the number of founders that would be expected to produce the same genetic diversity as the population under study if the founders were equally represented and no allele loss occurred:

Fe/fa and fg/fa ratios were estimated to determine genetic bottlenecks and random genetic drift, respectively.

Genetic contributions: The marginal contribution of each major ancestor “j” was calculated as proposed by Boichard et al. [39]. The genetic contributions were estimated for the top ten ancestors, with the maximum genetic impact between 1999 and 2023.

The CFC v.1.0 software was used to calculate ancestral contributions and gene origin probabilities [40].

### 2.8. Genetic Diversity

Genetic diversity (GD): The GD was estimated using the equation:

(1) GD=1−12fg

Genetic diversity loss (GD-loss): The GD-loss due to unequal contribution of founders was estimated according to Caballero and Toro [33,40] using 1 − GD*. The population GD-loss from the founder generation was estimated using 1 − GD:

(2) GD*=1−12fe

The difference between GD and GD* indicates the GD-loss due to genetic drift accumulated from the population founding [38], as well as the effective number of non-founders (Nenf). The unequal contribution of founders relates to the fact that the genetic contributions of founders of specific populations may be of different proportions due to past directional mating (human-mediated or not) during the process of population shaping.

### 2.9. Data Analysis and Software

The software used for the database analysis was ENDOG v. 4.8 [41], POPREP [42], and CFC [43] by means of which the demographic-derived parameters, genetic diversity indices, and gene origin probability were obtained.

## 3. Results

### 3.1. Population Structure and Reproductive Performance Evolution in Montbeliarde Cattle Obtained by Absorption Crossing

#### 3.1.1. Demographic Structure and Reproductive Performance

The population structure and reproductive performance evolution in Ecuadorian Montbeliarde cattle are shown in Table 1. While the historical percentage of sires and dams constituted about 15% and ~85%, respectively, these percentages increased to ~6.5% for sires and ~93.5% for dams in the 2019–2023 period, respectively. The trend from the first period analyzed (1999–2003) and the last period (2019–2023) was a decrease (−20.85%) in the number of individuals with pedigree, despite a peak in the number of individuals with pedigree in the third five-year period, 2009–2013. With regard to the number of individuals in the reference population, a decrease of ~59% was observed when the first and the fifth chronological periods were compared. Moreover, when the 2009–2013 period is compared to the last period, the reference population decreases by ~83%. In general, the total number of parents increased during the chronological periods, except for the last period, which shows a decrease in the total number of sires (−82%) but maintains the number of dams. With regard to the number of individuals with and without progeny, a decrease of 89% in individuals with progeny was detected when the first and fifth periods were compared, while a 12-fold increase in individuals without progeny was observed. Overall, the number of individuals just with known sire and dam, and those with no known (both) parents, was also substantially reduced over time. Thus, for the fourth period (2014–2018), all the individuals with known dams only were just 2, compared to 9 in the third period (2009–2013). Instead, the number of individuals with known sires only increased from 62 (1999–2013) to 104 (2019–2023). Most of the individuals were classified in the group of no known parents, which decreased from 45 (third period) to 5 (last period).

The analysis of the population census considered individuals born since 1999 (after the foundation of the Ecuadorian Montbeliarde Association). The previous (historical) records refer to the genealogical information of the ascendants. From this year onwards, there was sustained growth until 2013, after which the decline accelerated, reaching the year 2023 with only 22 individuals registered.

#### 3.1.2. Reproductive Performance per Sire and Dam

Regarding the reproductive parameters, the calvings per service ratio was maintained over time. On the other hand, the total number of calves born was reduced drastically over time (a decrease of >96%). After analyzing the average number of calves (offspring) per sire and dam, the average number decreased extremely from 6.53 to 0.21 (reduction of >96%) and from 16.43 to 3.00 (reduction of >81%), respectively, when the first and the last chronological period were compared. Analogously, a great reduction was observed regarding the maximum number of calves per sire and dam, decreasing from 79 to 5 (>93%) and from 5 to 2 (40%), respectively. Thus, the maximum progeny per sire (107) and dam (5) in the historical population decreased considerably compared to the last chronological period analyzed, where a decrease was observed to 5 calves per mated sire and slightly decreased also for mated dams to a value of 2, respectively, as shown in Table 1.

#### 3.1.3. Number of AI Services and Number of Calvings

**Figure 1:**
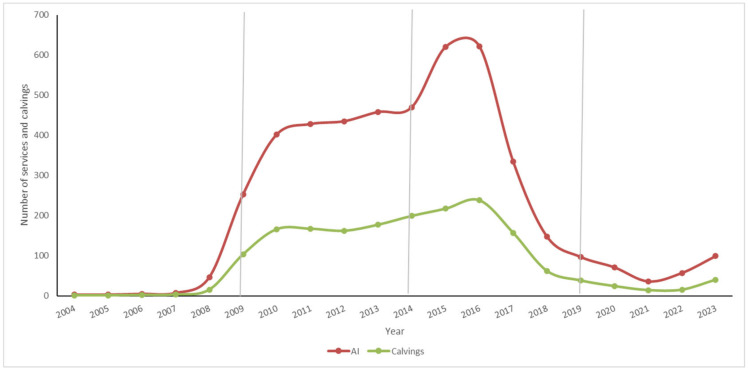
Evolution of number of services and number of calvings in a Montbeliarde cattle population obtained by absorption crossbreeding. AI: artificial insemination.

Figure 1 shows the evolution of the number of AI services and calvings in breeding Montbeliarde dairy cattle over time.

Artificial insemination (AI) was the most used Assisted Reproductive Technology (ART) over time, showing the highest record ever in the history of the Montbeliarde cattle breed in Ecuador in the 2014–2019 period compared to the other chronological periods; however, from 2016 onwards, it has had a downward trend until 2023. AI records were parallel to the number of calvings over time, which had a progressive upward trend (2009–2016). Finally, the use of AI has been relegated to a very low number of animals, remaining marginal compared to 2009–2016 (Figure 1).

#### 3.1.4. Evolution of the Number of Calvings

**Figure 2:**
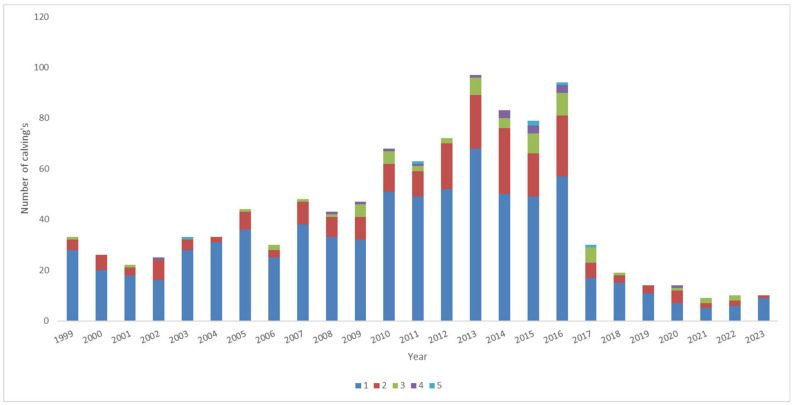
Distribution of dams by the number of calves/year (offspring) in a population of Montbeliarde cattle obtained by absorption crossing.

Figure 2 shows the evolution of the number of calvings in an Ecuadorian Montbeliarde cattle population over time (1999–2023).

The percentage of dams with one calving (blue: a calf per year) increased drastically (1999 to 2013) over time, while the percentage of dams with two (red), three (green), and four (purple) calves per year increased significantly until 2016. Moreover, during the last period (2019–2023), most of the dams showed three or fewer calves per year. From 2014 onwards (fourth period), coinciding with the implementation of embryo transfer-derived technologies and the application of AI in the Montbeliarde breed on a massive scale, the number of dams with more than three calves per year increased notably; however, from 2019 onwards (last five-year period), there were fewer dams showing more than three calves per year (Figure 2).

### 3.2. Pedigree Completeness and Generation Intervals in Ecuadorian Montbeliarde Cattle Obtained by Absorption Crossing

#### 3.2.1. Pedigree Completeness-Derived Parameters

Table 2 shows the pedigree completeness-derived parameters in the different populations. In general, a progressive decrease in PCI value was observed in all the chronological periods analyzed. The first-generation PCI value in the historical population was 72.95% and decreased to 65.87% in the period 2019–2023. The second- and third-generation PCI in the historical population was 61.79% and 47.07%, respectively, and in the period 2019–2023, it was 61.83% and 60.40%. In subsequent generations, there was a progressive decline in the PCI value, reaching 15.11% in the historical population (a reduction of 79% from the first to the fifth generation) and 45.45% in the fifth chronological period (a reduction of 32% from the first to the fifth generation in the 2019–2023 period).

On the other hand, the PCI decreased progressively between 1999 and 2023 in the first and second generations; however, this tendency changed from the third generation onwards, where the PCI increased progressively when the historical and the last chronological periods were compared. In some cases (e.g., within the fifth generation), the PCI increased three-fold from the first period until the fifth period studied. Finally, when GMax was analyzed between 1999 and 2023 in the Ecuadorian Montbeliarde cattle, this parameter increased more than two-fold in the last period (2019–2023) compared to the first five-year period. Similarly, the GEqu value increased almost two-fold when the same periods were compared. Regarding GCom, its value decreased over time until almost half of the value obtained in the first five-year period.

#### 3.2.2. Generation Intervals

**Figure 3:**
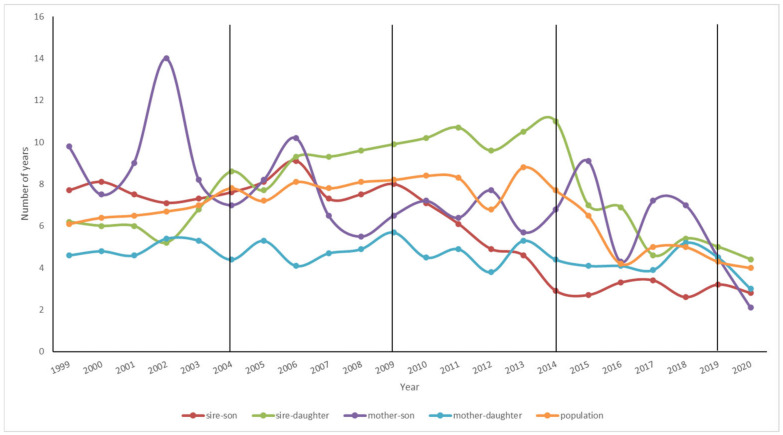
Generation interval (GI) evolution (1999 to 2023) regarding the four gametic pathways in Ecuadorian Montbeliarde cattle obtained by absorption crossing. The average GI value of the historical period was ~7 years, while in the most recent period studied (2019–2023), it was ~4 years.

Figure 3 shows the GI evolution in all gametic pathways (Sire-Son, Sire-Daughter, Dam-Son, and Dam-Daughter) over time in the Ecuadorian Montbeliarde cattle obtained by absorption crossing. The GI value was 7.17 and 3.08 years for the historical and the last (2019–2023) period, respectively. In the historical period, a great reduction in the GI value was observed when the Sire-Son and Sire-Daughter pathway was compared to the Dam-Son and Dam-Daughter pathway, with the former being much longer. Overall, regarding the GI value, there was a tendency to remain stable until the last two chronological periods. Then, a gradual decrease in the GI value was observed in the fourth and fifth periods. The GI value fluctuations were minimal over time regarding the Dam-Daughter gametic pathway. On the contrary, it is important to underline that the most accentuated differences regarding GI value were observed in the Dam-Son gametic pathway over time (Figure 3).

Moreover, with the arrival of genomic evaluation in Ecuador (since 2014 onwards), a considerable decrease in the GI value was observed in the last two periods analyzed (2014–2018 and 2019–2023) by the Sire-Son (3.92 years) and the Dam-Son gametic pathway (6.37 years). On the other hand, it was also observed that the Dam-Daughter pathway in the historical population was the lowest (4.18 years), occurring a slight increase (4.27 years) in the last chronological period 2019–2023. Finally, during the last two chronological periods, the GI value related to all gametic pathways decreased drastically (Figure 3).

#### 3.2.3. Inbreeding, Average Relatedness, Coancestry, and Non-Random Mating

Table 3 shows the results for average F, ΔF, maximum F, inbreeding, and highly inbred animals (%), C, AR, and GCI. The inbreeding coefficient was low (0.52%) in the historical population and higher (0.63%) in the last period studied. Only a low percentage of highly inbred animals was observed, with the maximum inbreeding coefficient being 26%.

**Figure 4:**
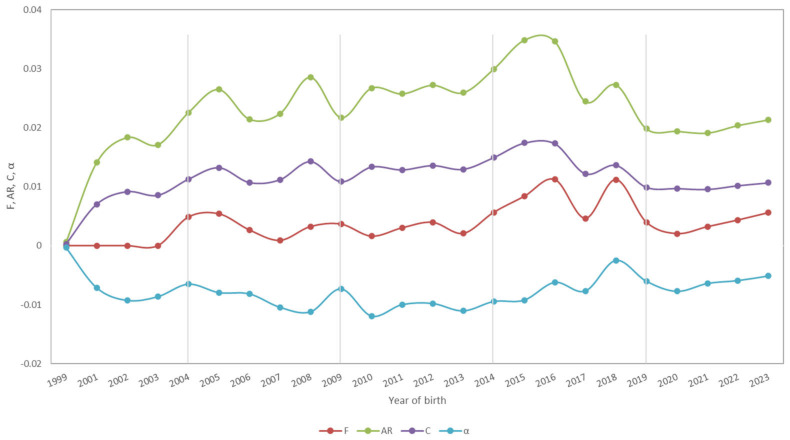
Inbreeding (F), average relatedness (AR), coancestry (C), and non-random mating (α) evolution (yearly) in Ecuadorian Montbeliarde cattle obtained by absorption crossing.

The historical value of the parameter inbred animals was ~27%, a value that decreased drastically to 2.38% at present. The inbreeding increment (ΔF) remained constant over time, having a peak value (0.24) in the period 1999–2003 (Table 3). Moreover, the percentage of highly inbred animals was 0.38% and 0.04% (historical vs. 2019–2023 period); the average coancestry coefficient was 1.29% and 1.03%, respectively. Average relatedness reached a maximum of 3.13% in the period 2014–2018, while the maximum average coancestry coefficient of 1.57% was reached in the same period (Figure 4). The GCI decreased from 3.45 in the historical population to 3.12 in the last period, with the highest historical peak observed in the period 2014–2018 with a value of 4.75 (Table 3).

The α value showed that individuals within the Montbeliarde population did not have the same probability of mating with any other individual, which may affect the Hardy-Weinberg equilibrium. The greatest α value was observed in 2018 (−0.0024) and the lowest in 2010 (−0.0119). The negative values indicated that the level of inbreeding in the Montbeliarde population was lower than expected. The more α negative the value, the lower the degree of mating between related individuals happened. An average value of α = −0.0077 indicated that the trend toward the value of 0 shows that related individuals were mating more frequently than appropriate (Figure 4).

#### 3.2.4. Effective Size (Ne)

**Figure 5:**
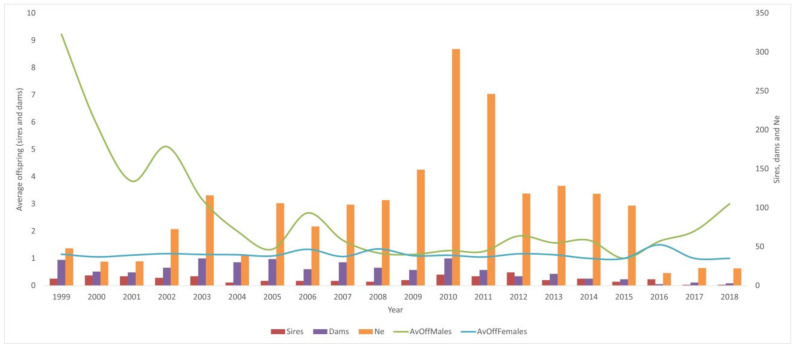
Effective size (Ne) evolution in Ecuadorian Montbeliarde cattle obtained by absorption crossing. Data from 2019 onwards were not available due to Ne was calculated using GI, and therefore, there is no information about the GI of the offspring (calves) born after 2019.

Figure 5 shows the evolution of the effective size (Ne) in Ecuadorian Montbeliarde cattle obtained by absorption crossing over time.

As stated in Figure 5, the average offspring (male and female calves) per sire/dam was quite stable from 2007 to 2015; however, after this year, the tendency of the males born increased together with a significant reduction of Ne of sires and dams. Given the drastic reduction in the number of sires and dams since 2015, a worrying decrease has been observed in the Ne value.

### 3.3. Gene Origin Probability, Ancestral Contributions, and Genetic Diversity in Ecuadorian Montbeliarde Cattle Obtained by Absorption Crossing

#### 3.3.1. Gene Origin Probability and Ancestral Contributions

The results of the analysis of the gene origin probability and ancestral contributions are shown in Table 4. The greatest number of founders contributing to the reference population was observed in the fourth period (n = 31); however, in the 1999–2003 period, a lower number of founders was observed (n = 174). The same trend was also observed for the fe and fg. Overall, after the genomic era (2010 onwards), the fa and fg decreased. On the other hand, the fe/fa and fg/fe ratios showed stability until 2009; however, the first one increased drastically from 2009 onwards, whereas the second one decreased progressively over time (Table 4). Thus, the fe/fa ratio indicated that the population has not remained stable over time. Therefore, it can be considered that, since 2009, individuals did not effectively contribute to the population. On the other hand, the ancestors explaining between 25%, 50%, and 75% were on average ~4, ~11, and ~37, respectively. In all cases, there was a progressive decline over time (Table 4).

Regarding the cumulative marginal genetic contribution (gene pool), the first 10 ancestors explained 45.95% of the gene pool in the historical population, while in the last population, they explained 40.75%. On the other hand, the individual genetic contribution of a single ancestor (Martien) explained 8.05% of the gene pool in the historical population while, in the last population, the sire called Gardian explained 6.35%. As for the ancestors contributing genetically to the population, Gardian, with 6.35% (1), Faucon, with 5.99% (2), and Micmac, with 5.95% (3), were coincident [44].

#### 3.3.2. Genetic Diversity

Table 5 shows the GD-derived parameters and the GD losses during the different chronological periods.

The main cause of GD loss in Montbeliarde cattle was shown to be genetic drift accumulated over non-founder generations, which is mainly caused by the small Ne value in all periods. An increase in the GD loss due to the bottleneck and genetic drift was estimated at 4.25%, with 0.55% due to the unequal contribution of founders. Overall, GD loss increased over time, being greatest in the last period (Table 5).

## 4. Discussion

The analysis of demographic structure and genetic diversity developed in the present study attempted to provide relevant information related to the breeding management and evolution of a Montbeliarde dairy cattle population obtained by absorption crossing. As stated, the Montbeliarde cattle were traditionally raised in Central European countries where the environmental conditions are radically different from those of a tropical latitude country. To the best of our knowledge, this was the first research to study the reproductive performance, inbreeding, and genetic diversity in a population of Montbeliarde dairy cattle raised in a South American country.

Regarding the analysis of the population census of the individuals born since 1999 (after the creation of the Ecuadorian Montbeliarde Association), a sustained growth until 2013 was observed, after which the population decline of this breed was intense. This effect was possibly due to the crisis that Ecuadorian dairy farmers suffered in recent years, especially because of the high production costs, fixed (uncontrolled) support prices, low per capita consumption, use of whey, and cattle rustling [45,46,47,48,49] together with the role of environmental factors at tropical high-altitude conditions and the interactions with genetic adaptation of this breed. Additionally, there was a considerable reduction in the proportion of sires and a drastic decrease in the proportion of dams, which directly affected the number of calves per sire and mated dam, respectively. This was probably due to the exclusive use and importation of AI doses from a very small number of Montbeliarde sires. On the other hand, in the case of dams, the population decrease could be due to the maintenance of the same reproductive and breeding practices, as well as the decision of producers to retain fewer females for replacement. An opposite effect was reported for the Braford beef breed, in which there was a greater interest in breeding males [50]. Recently, the ease of importing AI doses and cryopreserved embryos has increased their commercialization, which has had a notable influence on the application of ARTs by Montbeliarde cattle producers. In addition, the services provided by ART professionals have increased notably, decreasing the costs of the application of these technologies, as well as guaranteeing pregnancies. These factors, together, could again promote the recovery of this breed in Ecuador, decreasing production costs and generation intervals, on the one hand, and, on the other hand, increasing production yields by creating more profitable genetic lines.

With regard to the pedigree completeness, the number of historical generations traced for this breed obtained by absorption crossing was quite large when compared to the initial period (1999–2003). This was due to the fact that within the Montbeliarde cattle association, it was common practice to record information from their genealogical certificates (generally three generations of ancestors). This effect can also be seen in the fact that known ancestors through the paternal line in the 1999–2003 population (~70%) compared to known ancestors through the maternal line (~50%). Similarly, in all the populations analyzed, there was a progressive decrease in the level of pedigree completeness up to the 5th generation, reaching values of ~15% and ~45% for the historical population and that of the 2019–2023 period, respectively. This effect has also been observed in other dairy breeds, such as Holstein Friesian [51]. The accuracy and reliability of the population structure analysis depend on the pedigree completeness and the amount of genealogical information available between generations [52]. Additionally, MacCluer et al. [53] considered that a pedigree with a value higher than 0.6 related to the pedigree completeness produces reliable inbreeding estimates. In the case of the Montbeliarde breed, only the first and second generations showed values greater than 0.6 in the current population. On the other hand, similar values have been observed in other breeds, such as the Brangus in Argentina [54]. Likewise, the increase in equivalent generations (2.19 to 3.67), as in other cattle breeds such as the Brown Swiss (6.0 to 16.1) [55] or Japanese Black (5.1 to 10.2) [56], reflects that the pedigree quality has been improved, as well as the average number of known parents of the initial population compared to the last population. This fact was probably due to the management of an open studbook for the Montbeliarde breed and the fact that some individuals with no known parents have been registered. Therefore, to design effective future breeding programs, a great improvement with regard to the control of future registrations of cattle by the Montbeliarde Breeding Cattle Association would be needed. Secondly, there is a need to increase the future genealogical information by means of molecular techniques, such as genomics.

The Montbeliarde breed GI value decreased by half from the historical population until the most recent period (2019–2023). Anyway, the historical value of Ecuadorian Montbeliarde was greater than that obtained for the same breed in France (6.2 years) [57]. However, the GI value obtained in the latest period studied (2019–2023) was much lower than that obtained for the years 2021–2023 in France (5.3 years), according to other information sources [44,58]. Regarding the historical gametic pathways, a great reduction was observed when comparing the GI in the sire-son and sire-daughter with the dam-son and dam-daughter pathway, with the sire-related pathway being the greater. This trend was similar in some breeds such as the Simmental in Mexico and Colombia (8.5–6.5 years for the sire-son and sire-daughter; 5.9–5.4 years for the dam-son and dam-daughter pathway, respectively) [59], as well as in the Shorthorn breed in Japan (7.5–9.4 years for the sire-son and sire-daughter; 6.6–6.6 years for the dam-son and dam-daughter pathway, respectively) [60], and in the Holstein breed in Brazil (7.0–8.5 years for the sire-son and sire-daughter; 5.6–4.1 years for the dam-son and dam-daughter pathway, respectively) [51]. A long GI related to the sire-related pathway indicates that genetic material from older parents has been used in the Montbeliarde breed in Ecuador. For example, in individuals born in 2018, genetic material imported via seminal doses from sires born in France between 2002 and 2007 was observed. One of the reasons for the use of older sires was the reliability of the genetic tests. It was also quite influential that the sires have been maintained with superior genetic values within the breed, despite the high cost that the cattle producer was willing to pay. Generally, this problem occurs in countries where high-reliability semen was almost exclusively imported for genetic improvement of their cattle populations, that is, it has been progeny tested and widely used both nationally and internationally (e.g., INTERBULL evaluation) [51,61]. In conjunction with the advent of genomic valuation in the Montbeliarde breed of Ecuador (2014–2015), a considerable decrease in the GI was observed in the last periods (2014–2018 and 2019–2023) for the sire-son and dam-son pathways. On the other hand, it was also observed that the historical GI related to the dam-daughter pathway was the lowest, with a slight increase in the population studied during 2019–2023. Similar values have been reported in breeds such as the Holstein in Brazil, Spain, and the USA (4.1, 3.77, and 3.5 years, respectively) [51,62,63]. These values indicate that cattle breeders could be managing a high replacement rate of females within herds, considering that this can affect the productive life and profitability of the herd in general [63].

In the classification of herds according to the origin of use of the parents, there were no herds that acted as breeding nuclei. On the other hand, only one herd could be considered as a multiplier, and most herds were commercial herds because they exclusively use imported semen. The Montbeliarde breed was introduced into Ecuador from France through an absorption crossing scheme using artificial insemination. Only during the last chronological period had the embryos been used sporadically, when the breed was already established in terms of purity. Thus, the individuals considered purebred have been obtained through several generations using an absorption crossing scheme using artificial insemination doses from French purebred sires. This could be one of the reasons why no nucleus and almost no multipliers were found, being that the great majority of herds were of the commercial type. Similarly, in the Romosinuano breed introduced in Mexico, and in the Holstein Friesian breed introduced in Brazil, the same has been observed, with the exception of the number of multiplier herds, which was higher, indicating that a breed is growing with respect to its population [64,65].

Regarding the results obtained in the inbreeding-derived parameters (F, ΔF, Fmax, and highly inbred animals), a rather low average F value was observed in the historical and current populations. In addition, it was observed that about a quarter of the individuals were highly inbred. The average F value and matings between highly inbred animals (crosses between full and half siblings) reached maximum values at the beginning and during the application of the absorption crossing scheme during the 1990s, while the maximum values of coancestry (C and AR) were obtained during the period 2014–2018. With regard to the average α value (−0.0077), it was observed that in the Ecuadorian Monbeliarde, this value was possibly underestimated because when the absorption crossing scheme was applied, the dams were often not registered from the initial record; only the sire was officially registered, contrary to the case of the Berrenda Negra and Colorada [66]. Regarding the evolution of the GCI value, an increase over time was observed. The peak of inbreeding observed in the initial period (1999–2003) could be attributed to the recording of animals coming from the crossbreeding between individuals of the base population, as well as to the reduced number of sires used during this period. This effect was also found in the Holstein breed in south-eastern Brazil [51]. The current (2019–2023) average inbreeding of the Montbeliarde and the increase in inbreeding values were relatively higher than those obtained in the Ecuadorian Charolais [18]. This was possibly due to the fact that the Ecuadorian Charolais was recently conformed, structured, and established compared to the Ecuadorian Montbeliarde breed. However, the current Montbeliarde inbreeding values compared to those of other dairy breeds in other countries were quite low because the Ecuadorian Montbeliarde has not gone through a rigorous process of genetic improvement, despite the fact that the first official records made on this breed have been present since the late 1990s. For example, the Montbeliarde breed in France, from which it originates, had an average inbreeding rate of 5.5% in the last 10 years [44]. In the case of other breeds, such as the Holstein, Jersey, and Brown Swiss, the inbreeding values were also higher [51,67]. Similarly, the inbreeding values were also higher in the local Guzerat, tropical dairy Creole, and Gyr, among others [68,69,70]. This effect could also be due to the fact that pedigree integrity is still low in the current Montbeliarde maternal pathway raised in Ecuador. On the other hand, the Ne value has been decreasing over time, which has been considered a very dangerous value for the preservation of the breed, especially during the last 10 years. The recommended value for Ne has been established in at least 50 individuals according to FAO [71]; however, this Ne value has been observed to be far from the recent Ne value, which is around 16–22 individuals when sires and dams were considered.

Regarding the results related to the analysis of the gene origin probability obtained in the present study, specifically, the fe/fa value indicated that the Montbeliarde population has remained stable when considering the individuals that effectively contribute to the whole population. Thus, the fe/fa ratio estimated in the current Montbeliarde population of Ecuador was similar to the French population, and this fact could be due to the great majority of the Ecuadorian population possessing ancestors of French origin [44]. On the other hand, regarding the results obtained in the ancestral GD contribution (accumulated marginal genetic contribution), the main ancestor contributed around 10% GD, with a total of 10 ancestors explaining 50% of the historical GD in the Ecuadorian Montbeliarde breed. On the other hand, the GD contribution in the French Montbeliarde population was around 15%, being that only 6 ancestors explained the same historical GD [44]. This fact could be due to the fact that the pedigree completeness was higher in the French Montbeliarde population compared to the Ecuadorian population, as well as the number of descendants [44].

Regarding GD, the estimated value for this breed was lower compared to different native breeds in Spain, such as the Negra Andaluza [18], Berrenda Negra, and Colorada [66], or even other native beef breeds in Slovakia [72], as well as other improved zebu breeds, such as the Gyr, Nelore, and Brahman in Brazil [73]. Genetic drift is a non-directional process that causes loss of genetic variation in populations, genetic differentiation between populations, and increased homozygosity. It is now believed that large populations of individuals have been subject to genetic drift through bottleneck or founder effects [74]. The GD loss in all periods studied marked an increasing trend, except for the period 2005–2008, and the highest GD loss occurred in the last period (2019–2023). GD in the reference population considered for GD loss due to the unequal contribution of founders showed a significant increase, whose percentage decreased markedly.

While the GD loss value doubled due to genetic drift-derived effects, the GD loss due to bottleneck and genetic drift from founders increased by more than 60%. This indicates that the GD loss due to genetic drift and bottleneck was greater than the GD loss due to unequal contribution from founders. Such an effect was similar to that reported by Kadlečík et al. [72] for Slovakian beef breeds. Moreover, this effect could be due to the reduced Ne value, as the population registered in the Ecuadorian Montbeliarde Association has been drastically reduced in recent years, as well as the decrease in demand for semen straw imports in this breed. Because of these factors, the number of sires currently available belong to only two importing companies that market insemination doses belonging to about 20 sires of the Monbeliarde breed (without knowing their degree of relatedness) compared to the 278 sires of the Holstein Friesian breed marketed by these two companies [75]. The effect of GD loss by genetic drift accumulated over non-founder generations has been reported in several breeds, such as the Holstein Friesian and Jersey [76] and Milking Shorthorn [77], in which a small Ne value indicated an intensive use of a few sires of high genetic value. A similar effect was observed in the Negra Andaluza breed, which has gone through several bottleneck processes such as agricultural mechanization, crossbreeding with other breeds, lack of genetic improvement programs, segmentation of subpopulations, and unstructured breeding efforts that contributed to population reduction and GD loss [18,78]. Similarly, the Ecuadorian Monbeliarde has gone through several similar processes, including crossbreeding to improve its adaptation, especially to high altitude animal production systems (>3200 m.a.s.l.), where the ultraviolet rays have possibly affected its vision system since this breed and others such as Simmental and Normande have depigmented skin around eyes and periorbital tissues, which facilitates the development of carcinomas [79]. These environmental constraints and their interactions with genetic adaptation are a great challenge faced by Montbeliarde cattle in Ecuador. This could have generated an effect on cattle breeders, who have desisted from breeding the Montbelairde breed, as observed in the results of the present study. On the other hand, it was also observed that a single ancestor contains >13% of the GD, similar to the prediction of 15% reported for the year 2024 by IDELE [44]. Thus, the number of ancestors explaining 50% of GD in Ecuador was 10 compared to 6 in France. These values could be related to a reduction in the population of females, births, and ancestors. For example, the inbreeding rates of 41.9% of the French population were between 6.25–12.5%. To avoid this GD loss, it would be necessary to perform breeding schemes considering a low coancestry value in order to maximize GD in future generations, as recommended by Lacy [80] and Caballero and Toro [33]. Thus, low Ne increased the spread of inherited disorders and the reduction in fitness associated with GD loss [71]. According to the FAO, a 1% increase in inbreeding was associated with a decrease of 0.4–0.6% of the phenotypic mean for milk, fat, and protein yields and an increase of 0.02–0.05% for calving intervals [71]. Thus, the GD loss can be managed either by minimizing overall inbreeding within the breeding scheme or by targeting specific regions of the genome associated with inbreeding depression [71]. Therefore, reducing genetic drift, such as optimizing mating strategies or introducing unrelated sires from external populations, would resolve, at least in part, the problem related to GD loss in Montbeliarde cattle.

## 5. Conclusions

In summary, the results indicated that inbreeding rates and GD losses were accumulating linearly and were very close to critical levels, which can result in negative effects on breeding and conservation purposes. The greater part of the GD reduction can be attributed to genetic drift that accumulated in the non-founder generations, indicating the disproportionate influence of non-founders due to the historical and recent reduction of the effective population size together with the intensive use of a few elite AI sires. Intense strategies to maintain GD in the Montbeliarde population must be implemented, such as increasing the number of AI sires with a low individual inbreeding coefficient and increasing the effective population size. Finally, this study indicated the necessity for active mating strategies and inbreeding and coancestry rate management to preserve the GD and ensure the long-term sustainability of Montbeliarde cattle.

## Figures and Tables

**Table 1:** Population structure and reproductive performance-derived parameters in Ecuadorian Montbeliarde cattle from 1999 to 2023.

Demographic Parameter	Historical	1999–2003	2004–2008	2009–2013	2014–2018	2019–2023
Number of animals with pedigree	2399	211	332	747	434	167
Number of animals (reference population)	1309	141	198	338	308	58
Dams (total)	2033	151	296	681	392	156
Sires (total)	366	60	36	66	42	11
Individuals with progeny (offspring)	1320	199	215	310	94	22
Individuals without progeny (offspring)	1079	12	117	437	340	145
Number of animals with both known parents	1309	141	198	338	308	58
Number of animals only with known sire	868	62	125	355	123	104
Number of animals only with known dam	14	-	2	9	2	-
Number of animals with no known parents	208	8	7	45	1	5
**Reproductive parameter**		**1999–2003**	**2004–2008**	**2009–2013**	**2014–2018**	**2019–2023**
Calvings per service (AI) ratio (%)	39.27	-	34.38	39.24	39.77	36.39
Number of calves born (offspring)	3500	986	431	500	211	33
Average number of calves per sire	1.72	6.53	1.46	0.73	0.54	0.21
Maximum number of calves per sire	107	79	54	15	29	5
Average number of calves per dam	9.56	16.43	11.97	7.58	5.02	3.00
Maximum number of calves per dam	5	5	5	5	3	2

**Table 2:** Pedigree completeness-derived parameters in a population of Montbeliarde cattle obtained by absorption crossing.

Parameter	Historical *	1999–2003	2004–2008	2009–2013	2014–2018	2019–2023
**Population of animals with pedigree**	2399	211	332	747	434	167
**Number of generations (n)**	13	7	8	10	11	13
**1st generation (%)**	72.95	81.52	78.77	69.61	85.37	65.87
**2nd generation (%)**	61.79	70.26	70.63	63.69	77.07	61.83
**3rd generation (%)**	47.07	44.67	53.09	50.69	69.64	60.40
**4th generation (%)**	28.23	19.82	30.23	28.75	48.44	54.83
**5th generation (%)**	15.11	2.61	7.93	15.37	30.39	45.45
**Average GMax**	4.70	3.94	4.58	4.79	6.34	9.22
**Average GCom**	0.91	1.08	1.00	0.80	1.35	0.65
**Average GEqu**	2.36	2.19	2.42	2.32	3.30	3.67

GMax: Maximum number of generations; GCom: Complete number of generations; GEqu: Equivalent number of generations; * the historical period included individuals/data before 1999.

**Table 3:** Inbreeding (F), average relatedness (AR), coancestry (C), and non-random mating (α) parameters in Ecuadorian Montbeliarde cattle obtained by absorption crossing.

Parameter	Historical	1999–2003	2004–2008	2009–2013	2014–2018	2019–2023
Inbreeding coefficient (F, %)	0.52	0.63	0.57	0.46	0.90	0.63
Inbreeding increment (ΔF, %)	0.15	0.24	0.17	0.13	0.22	0.12
Maximum inbreeding coefficient (%)	26.56	25.00	25.00	26.56	7.62	26.12
Inbred animals (%)	26.72	1.58	4.04	8.05	9.84	2.38
Highly inbred animals (%)	0.38	0.13	0.04	0.08	0.00	0.04
Coancestry coefficient (C, %)	1.29	1.36	1.40	1.34	1.57	1.03
Average relatedness (AR, %)	2.58	2.72	2.80	2.68	3.13	2.06
Genetic conservation index (GCI)	3.45	3.57	3.82	3.37	4.75	3.12

**Table 4:** Gene origin probability and ancestral contributions in Ecuadorian Montbeliarde cattle obtained by absorption crossing.

Gene-Origin/Ancestral Contribution Parameters	Population	1999–2003	2004–2008	2009–2013	2014–2018	2019–2023
Historical population (n)	2399	211	332	747	434	167
Reference population (n)	1309	141	198	338	308	58
Base population (one or more unknown parents)	1090	8	7	45	1	5
Current base population (one unknown parent = half founder)	649	62	127	364	125	104
Number of founders contributing to the reference population (n)	459	174	256	330	351	231
Number of ancestors contributing to the reference population (n)	439	145	202	273	228	52
Effective number of non-founders (Nenf)	34.82	28.49	28.63	25.71	20.62	13.52
Effective number of founders (fe)	71.64	59.21	64.49	66.06	68.69	90.64
Effective number of ancestors (fa)	37	31	33	31	28	23
Founder genome equivalents (fg)	23.43	19.24	19.83	18.51	15.86	11.76
fe/fa ratio	1.94	1.91	1.95	2.13	2.45	3.94
fg/fe ratio	0.33	0.32	0.31	0.28	0.23	0.13
Number of ancestors to explain:						
25% of gene pool	4	4	4	4	4	3
50% of gene pool	14	11	11	11	9	10
75% of gene pool	60	38	46	43	37	22
100% of gene pool	145	202	273	228	52	145

**Table 5:** Genetic diversity-derived parameters in Ecuadorian Montbeliarde cattle obtained by absorption crossing.

Genetic Diversity Parameters	1999–2003	2004–2008	2009–2013	2014–2018	2019–2023	% COT
GD (%)	97.40	97.48	97.30	96.85	95.75	1.35
1 − GD (GD loss)	2.60	2.52	2.70	3.15	4.25	1.65
GD* (%)	99.16	99.22	99.24	99.27	99.45	0.29
Proportion of unequal contributions of the founders in GD loss (%)	0.84	0.78	0.76	0.73	0.55	−0.29
Proportion of random genetic drift in GD loss (%)	1.75	1.75	1.94	2.42	3.70	1.95
Proportion of random genetic drift and bottle necks in GD loss (%)	2.60	2.52	2.70	3.15	4.25	1.65

GD: Genetic diversity. The probability of gene origin given by the effective number of founders; GD*: Genetic diversity in the reference population considered to compute the genetic diversity loss due to the unequal contribution of founders; the effective number of founders (fe) and founder genome equivalents (fg). % COT: percentage of change over time.

## Data Availability

Data will be made available from the corresponding author upon reasonable request.
